# Correlation of Serum Zinc Levels with Hepatic Encephalopathy Severity in Patients with Decompensated Liver Cirrhosis: A Prospective Observational Study from Egypt

**DOI:** 10.1007/s12011-025-04544-x

**Published:** 2025-02-13

**Authors:** Atteyat A. Semeya, Rasha Elgamal, Amira A. A. Othman

**Affiliations:** 1 Hepatology, Gastroenterology and Infectious Diseases Department, Benha Teaching Hospital, Benha, Egypt; 2 Clinical Pathology Department, Faculty of Medicine, Suez University, Suez, Egypt; 3 Internal Medicine Department, Faculty of Medicine, Suez University, Suez, 43511 Egypt

**Keywords:** Albumin, Chronic liver disease, Decompensated liver cirrhosis, Hepatic encephalopathy, Zinc

## Abstract

A vital trace element, zinc, is involved in several metabolic and enzymatic functions, such as antioxidant defense and ammonia detoxification. Zinc metabolism is disturbed by liver cirrhosis, especially when it is decompensated, contributing to systemic complications, including hepatic encephalopathy (HE). This study aimed to assess serum zinc levels in patients with decompensated liver cirrhosis and evaluate their correlation with the severity of cirrhosis and HE grades. This prospective observational study included 100 patients with decompensated liver cirrhosis and 100 healthy controls between December 2022 and June 2023. Serum zinc levels and other biochemical parameters were measured using standard laboratory methods. Liver cirrhosis severity was evaluated using the Child–Pugh score, and HE was graded using the West Haven criteria. Correlations between zinc levels, clinical parameters, and disease severity were analyzed statistically by Spearman’s correlation and Kruskal–Wallis tests. Serum zinc levels were significantly lower in cirrhotic patients compared to controls (21.7 ± 24.3 µg/dL vs. 85.9 ± 32.6 µg/dL, *P* < 0.0001). Zinc levels inversely correlated with both Child–Pugh class (*r* =  − 0.84, *P* < 0.001) and HE grade (*r* =  − 0.78, *P* < 0.001). Patients with advanced Child–Pugh Class C or HE Grade 3 had severe zinc deficiency. A strong positive correlation was observed between serum zinc and albumin levels (*r* = 0.843, *P* < 0.0001), underscoring albumin’s role in zinc transport. Serum zinc deficiency is strongly correlated with the severity of liver cirrhosis and HE. Therefore, routine zinc assessment and supplementation should be considered in cirrhotic patients, especially those with hypoalbuminemia or advanced HE for better outcomes.

## Introduction

Liver fibrosis, resulting from chronic conditions like hepatitis and fatty liver disease, leads to cirrhosis, where progressive scarring impairs liver function. Decompensated cirrhosis introduces complications such as ascites, gastrointestinal bleeding, and hepatic encephalopathy (HE), a major cause of morbidity and mortality [1, 2]. Globally, cirrhosis is a leading cause of death, accounting for 3.5% of all fatalities [3], with Egypt historically facing a high burden due to hepatitis C virus (HCV) prevalence despite recent public health efforts to reduce it [4–6].

Hepatic encephalopathy (HE) is reversible brain dysfunction occurring in hepatic insufficiency and/or portosystemic shunting. It can manifest as a wide spectrum of neurological or psychiatric abnormalities ranging from subclinical brain dysfunction to coma [7, 8]. Studies indicate that hepatic encephalopathy (HE) affects approximately 30–45% of individuals with cirrhosis. Within the first 5 years following cirrhosis diagnosis, there is a 5–25% probability of experiencing a first episode of HE; in contrast, those who have experienced overt HE in the past are at a 40% risk of undergoing a 1-year recurrence [9]. The pathogenesis of hepatic encephalopathy (HE) is not fully understood, but it is primarily caused by portosystemic shunting and hepatocellular failure. Ammonia, a gut-derived toxin, escapes hepatic detoxification due to liver disease, leading to low-grade cerebral edema that impairs neuronal function. Early diagnosis and intervention can significantly improve outcomes, particularly in patients with minimal or overt HE. However, if the underlying liver disease remains untreated or progresses, HE may recur or become more challenging to manage [10].

In Egypt, decompensated liver cirrhosis is primarily caused by chronic hepatitis C virus (HCV) infection, which historically had one of the highest prevalence rates globally due to unsafe medical practices, leading to a significant burden of chronic liver disease and subsequent progression to cirrhosis. Hepatitis B virus (HBV) infection remains another important cause, though less prevalent than HCV [6]. Non-alcoholic fatty liver disease (NAFLD) and metabolic dysfunction-associated fatty liver disease (MAFLD) are also emerging as major contributors, driven by increasing rates of obesity and type 2 diabetes [11]. While alcohol-related liver disease is less common in Egypt due to cultural and religious practices, it still contributes to the overall burden [12]. These etiological factors underscore the multifaceted nature of decompensated cirrhosis in Egypt and highlight the need for integrated public health strategies targeting viral hepatitis, metabolic liver disease, and other contributing factors.

The liver governs trace element metabolism, transport, bioavailability, tissue distribution, and ultimately excretion through bile production [13]. Trace elements are required for supporting important activities, and excess or shortage causes metabolic diseases [14]. Decompensated cirrhosis often alters the metabolism of trace elements such as zinc, copper, manganese, and magnesium resulting in malnutrition and micronutrient deficiencies [15]. There is a strong link between liver disease presence and progression and the metabolism of trace elements [14]. Zinc is an essential co-factor in DNA synthesis, an anti-inflammatory, an anti-apoptotic, a membrane and cytoskeletal stabilizer, an antioxidant agent, etc. [16]. More than 300 enzymes have recently been known to use zinc as an active center or cofactor [17]. The primary plasma carrier of zinc is albumin, and serum albumin availability affects how much zinc is carried in the blood in addition to zinc itself [18]. Because of the impairment of nitrogen metabolism by reducing the activity of urea cycle enzyme, ornithine transcarbamylase in the liver, and glutamine synthetase in the muscle, zinc deficiency in cirrhotic patients has been associated with hepatic steatosis, impaired wound healing, dysgeusia, and hepatic encephalopathy [19]. In patients with cirrhosis of the liver, increased blood ammonia levels are inversely associated with serum zinc levels [20]. Zinc deficiency can impair the conversion of ammonia to urea in the liver, leading to elevated systemic ammonia levels [21]. This relationship highlights the potential role of zinc deficiency in the pathogenesis and exacerbation of hepatic encephalopathy, as impaired ammonia metabolism further contributes to neurotoxicity and neuronal dysfunction in cirrhotic patients [22].

Our study aimed to estimate serum zinc levels in all patients with liver cirrhosis with HE and to find out the correlation between serum zinc levels and (1) the severity of liver cirrhosis determined by Child–Pugh classification and (2) the development and severity of hepatic encephalopathy grades by West Haven classification.

## Subjects and Methods

### Study Population and Design

This prospective observational study was conducted at the Banha Teaching Hospital’s Department of General Medicine between December 2022 and June 2023. The sample size was calculated using the Cochrane formula for prevalence studies, considering a Z-score of 1.96 at a 95% confidence interval, a significance level of 0.05, and an estimated prevalence of 9% based on a previous study [23]. The minimum required sample size was approximately 126 participants. To ensure adequate power for comparisons, a total sample size of 200 participants was chosen, divided equally into two groups: group 1, enclosed 100 individuals without a history of liver disease (control group), with a mean age of 54.3 ± 14.2 years (they were 38 female (38%) and 62 male (62%)); and group 2, enclosed 100 individuals with decompensated liver cirrhosis and hepatic encephalopathy, (cases group), with a mean age of 62.2 ± 10.4 years (they were 43 female (43%) and 57 male (57%)). The inclusion of a control group was crucial for establishing baseline serum zinc levels in healthy individuals. This comparison enabled us to quantify the degree of zinc deficiency in cirrhotic patients with hepatic encephalopathy (HE) and to assess how liver dysfunction impacts systemic zinc metabolism. By providing a reference point, the control group strengthens the study’s ability to demonstrate the relationship between zinc levels, liver dysfunction, and HE severity.

### Ethical Considerations

The current study was implemented in coordination with the guidelines of the Declaration of Helsinki. Ethical approval was gained according to the recommendations of Ethics Unite of Banha Teaching Hospitals, Egypt, Approval #: HB-000122. Informed consent was obtained from the patients, which addressed all the steps of the study and their right to withdraw at any time.

### Inclusion Criteria

Adult patients of both sexes, aged > 18, who had given written informed consent and were diagnosed with decompensated cirrhosis of the liver using an abdominal ultrasound along with a clinical and biochemical assessment were included in the current study. The controls were healthy individuals with no history of liver disease.

### Exclusion Criteria

The study excluded pregnant women, patients with mental disorders or altered sensorium due to head injury or stroke, patients with other metabolic causes of encephalopathy, those in alcohol withdrawal or with acute alcohol intoxication, patients with acute or chronic diarrhea or malabsorption syndrome, individuals taking zinc supplements for any reason, patients with acute or chronic inflammatory conditions unrelated to chronic liver disease, and those with renal failure or malignancy.

### Clinical Assessment

All subjects were subjected to full history taking, and thorough physical examination, and demographic and clinical data, including age, sex, medical history, and cirrhosis etiology, were recorded. The severity of liver cirrhosis was assessed using the Child–Pugh classification, incorporating five parameters: prothrombin time (PT)/international normalized ratio (INR), ascites, encephalopathy, serum bilirubin, and serum albumin [24]. The presence and severity of hepatic encephalopathy were determined using the West Haven classification, which distinguishes four clinical grades [15]. Grade I is characterized by subtle personality changes and impaired attention, often noticed by family members. Grade II includes disorientation to time, inappropriate behavior, and lethargy. In Grade III, patients are stuporous but responsive to stimuli, exhibiting confusion and abnormal behavior. Grade IV is marked by coma [25].

### Laboratory Investigations

Five milliliters (mL) of fresh venous blood was collected from each participant after an overnight fast, with 2.5 mL of ethylenediaminetetraacetic acid (EDTA) for complete blood count (CBC) and glycated hemoglobin (HbA1c), and 2.5 mL drawn without anticoagulants for other serum testing. The samples were then centrifuged at 3000 rpm for 15 min, and serum aliquots were stored at − 20 °C until further analysis. All samples were promptly sent to the laboratory, where serum tests for lipid profile (total cholesterol, triglycerides, low-density lipoprotein, high-density lipoprotein), liver function (alanine aminotransferase, aspartate aminotransferase, total and direct bilirubin, albumin), kidney function (creatinine, urea), electrolytes (sodium, potassium, calcium), and fasting blood sugar were performed using the automated Cobas c 111 analyzer (Roche Diagnostics). The CBC and HbA1c were analyzed using the Sysmex XN-550 Cell Counter. Additionally, serum zinc levels were measured using atomic absorption spectrometry.

The “Japanese Society of Clinical Nutrition” defines serum zinc levels of ≥ 80 µg/dL as normal, 60 − 80 µg/dL as marginal deficiency, and levels < 60 µg/dL as clinical deficiency [26]. On the other hand, “Harrison’s Principles of Internal Medicine,” a widely recognized international reference, classifies zinc levels of < 70 µg/dL as low serum zinc concentrations and ≥ 70 µg/dL to indicate normal serum zinc concentrations [27].

### Statistical Analysis

Statistical analyses were performed using SPSS for Windows, Version 28 (SPSS Inc., Chicago, IL, USA). Quantitative data were expressed as means ± standard deviation (SD), while qualitative data were presented as numbers and percentages. The chi-square test (χ^2^) was used to compare qualitative (categorical) variables. For normally distributed continuous data, the Student’s *t*-test was applied to compare mean values between the two groups. Correlations between serum zinc levels and clinical/biochemical parameters were assessed using Pearson’s correlation analysis for normally distributed data and Spearman’s rank correlation test for skewed data. Comparisons of serum zinc levels across multiple groups, such as hepatic encephalopathy (HE) grades and Child–Pugh classes, were conducted using the Kruskal–Wallis H test due to the non-normal distribution of the data. Correlations between serum zinc levels, the severity of liver cirrhosis (Child–Pugh score), and the severity of hepatic encephalopathy (West Haven grade) were assessed using Spearman’s rank correlation test. A *p*-value < 0.05 was considered statistically significant.

## Results

### Baseline Characteristics

Patients ranged in age from 35 to 80 years old, with a mean age of 62.2 ± 10.4 years. Over one-third of the participants were > 65. Age and sex were not significantly different between patients and controls (*P* > 0.05), indicating that both groups were demographically comparable. This comparability is critical for ensuring that observed differences in biochemical parameters or disease severity are not due to age or sex differences. Hence, any variations in clinical outcomes can be more confidently attributed to liver dysfunction and zinc deficiency (Table 1). Most liver cirrhosis cases (78.0%) were attributed to hepatitis C infection, followed by bilharzia infection (14.0%) and other causes (9.0%).

**Table 1:** Baseline characteristics between cases and controls

Variable	Controls (*N* = 100)	Patients (*N* = 100)	*P*-value
Sociodemographic data			
Age (years) Sex (male/female)	54.3 ± 14.2 62/38	62.2 ± 10.4 57/43	0.472 0.067

Patients (*n* = 100), presented with varied and multiple complaints simultaneously. The most common presenting symptom was abdominal distension in 72% of cases, followed by pedal edema in 66%, icterus in 61%, fever in 51%, constipation in 49%, disorientation in 47%, altered sleep patterns in 46%, asterixis in 44%, melena in 43%, hematemesis in 40%, abdominal pain in 39%, nausea and vomiting in 24%, confusion in 16%, and diarrhea in 4%. Coma was observed in 8% of cases.

### Biochemical Parameters

Multifaceted biochemical changes in patients with decompensated liver cirrhosis compared to controls were provided (Table 2).

**Table 2:** Comparison of biochemical parameters between cases and controls

Variable	Controls (*N* = 100)	Patients (*N* = 100)	*P*-value
Liver functions			
ALT (IU/L)	39.1 ± 38.6	68.1 ± 95.8	**0.050**
AST (IU/L)	36.7 ± 23.9	70.3 ± 61.8	** < 0.0001**
Total bilirubin (mg/dL)	2.6 ± 2.3	4.6 ± 4.5	**0.0001**
Direct bilirubin (mg/dL)	1.2 ± 1.5	2.3 ± 3.7	**0.006**
Albumin (g/dL)	3.5 ± 1.4	2.7 ± 0.5	** < 0.0001**
Kidney functions			
BUN (mg/dL)	29.7 ± 10.2	36.4 ± 9.0	**0.026**
Creatinine (mg/dL)	0.94 ± 0.3	1.81 ± 0.2	**0.0004**
Hematological parameter			
HB (g/dL)	12.3 ± 1.9	10.4 ± 2.7	** < 0.0001**
PLT (× 10^3^/mm^3^)	139.1 ± 113.8	109.4 ± 78.3	**0.033S**
TLC	5.2 ± 2.8	4.9 ± 2.1	0.392
Coagulation profile			
PT (Seconds)	13.3 ± 3.2	19.6 ± 4.8	** < 0.0001**
INR	1.2 ± 0.3	1.6 ± 0.4	** < 0.0001**
Electrolytes			
Sodium (mEq/L)	137.9 ± 3.0	133.1 ± 5.2	** < 0.0001**
Potassium (mEq/L)	4.4 ± 0.7	4.1 ± 0.7	**0.003**
Calcium total (mg/dL)	8.6 ± 0.7	8.3 ± 0.7	**0.003**
Blood glucose profile			
FBS (mg/dL)	101.8 ± 41.8	97.1 ± 27.7	0.349
HbA1C (%)	5.4 ± 1.9	5.1 ± 1.5	0.217
Lipid profile			
Cholesterol (mg/dL)	125.7 ± 37.9	134.0 ± 52.8	0.203
Triglyceride (mg/dL)	92.2 ± 45.5	91.2 ± 56.8	0.891
HDL (mg/dL)	37.9 ± 13.3	48.9 ± 55.7	**0.046**
LDL (mg/dL)	70.3 ± 31.8	83.7 ± 48.7	**0.022**
Zinc level (µg/dL)	85.9 ± 32.6	21.7 ± 24.3	** < 0.0001**

*ALT* alanine aminotransferase, *AST* aspartate transaminase, *BUN* blood urea nitrogen, *HB* hemoglobin, *PLT* platelets, *TLC* total leucocyte count, *PT* prothrombin time, *INR* international normalized ratio, *FBS* fasting blood sugar, *HbA1C* hemoglobin A1C. A *P* < 0.05 = significant; *P* < 0.01 = highly significant

Liver function parameters such as significantly elevated AST (*P* < 0.0001) and bilirubin (total and direct) (*P* < 0.0001) reflect hepatocellular damage and impaired bilirubin excretion, while reduced significantly albumin (*P* < 0.0001) indicates decreased liver synthetic function, contributing to ascites and edema. Additionally, *P* = 0.050 for ALT indicates potential liver injury in both groups, though the evidence is borderline than to other liver profile parameters.

The Kidney Profile, including blood urea nitrogen (BUN) and creatinine, provides insights into renal function and the impact of cirrhosis on the kidneys. BUN (*P* = 0.026) showed a significant increase in patients compared to controls. Creatinine (*P* = 0.0004) was significantly higher in patients, indicating potential renal dysfunction as a result of impaired renal perfusion and reduced filtration capacity due to cirrhosis-associated complications.

Hematological changes, such as significant thrombocytopenia (*P* = 0.033) and anemia (*P* < 0.0001), result from splenic sequestration, chronic blood loss, and nutritional deficiencies. Total leukocyte count (TLC), with a *P*-value of 0.392, did not show a statistically significant difference between patients and controls. However, low TLC may indicate an overall suppressed immune function in cirrhosis, especially in the context of chronic disease, infections, or splenomegaly, which is common in advanced cirrhosis. Coagulation abnormalities, including significant prolonged PT (*P* < 0.0001) and significant elevated INR (*P* < 0.0001), indicate impaired clotting factor production and increasing bleeding risk.

Electrolyte imbalances, including significantly lower sodium (Na^+^) (*P* < 0.0001), potassium (K^+^) (*P* = 0.003), and calcium (Ca^2+^) (*P* = 0.003), are attributed to fluid retention, diuretic use, and impaired renal handling, exacerbating complications like hepatic encephalopathy. Blood glucose parameters, including fasting blood glucose (FBG) (*P* = 0.217) and HbA1c (*P* = 0.705), did not show a statistically significant difference between patients and controls, suggesting that, at least based on these parameters, hepatic diabetes or significant insulin resistance might not be apparent in this study.

The lipid profile shows reduced cholesterol, LDL, and HDL, reflecting impaired hepatic lipid metabolism and disease severity, although the changes in cholesterol and triglycerides were not statistically significant (*P* = 0.203 and *P* = 0.891, respectively). Lipid metabolism is significantly altered in cirrhotic patients due to impaired liver function, which affects both lipid synthesis and lipid clearance. Importantly, serum zinc levels are significantly lower in patients (*P* < 0.0001) in comparison to controls. The average serum zinc level for the patients was 21.7 ± 24.3 µg/dL, whereas the controls had a mean of 85.9 ± 32.6 µg/dL.

### Correlation Between Serum Zinc Levels and Clinical Parameters

The correlation between serum zinc levels and clinical/biochemical parameters was provided (Table 3). A strong positive correlation was observed between serum zinc and albumin levels, indicating the critical role of albumin in transporting zinc in plasma. Monitoring albumin levels may help in evaluating zinc status in cirrhotic patients. Other parameters, including age, bilirubin, AST, ALT, total bilirubin, creatinine, urea, HB, platelet count, PT, sodium, potassium, and calcium total did not show significant correlations, thus did not influence zinc levels.

**Table 3:** Correlation of serum zinc levels with clinical and biochemical parameters

Variables	Correlation (r)	*P*-value
Age	0.217	0.319
AST (IU/L)	− 0.077	0.727
ALT (IU/L)	0.102	0.644
Albumin (g/dL)	0.843	** < 0.0001**
Total bilirubin (mg/dL)	0.253	0.244
Creatinine (mg/dL)	− 0.238	0.273
BUN (mg/dL)	− 0.014	0.949
HB (g/dL)	− 0.084	0.703
PLT (× 10^3^/mm^3^)	− 0.187	0.393
PT (seconds)	0.305	0.157
Sodium (mEq/L)	− 0.092	0.671
Potassium (mEq/L)	− 0.065	0.769
Calcium total (mg/dL)	− 0.095	0.802
Zinc level (µg/dL)	-	-

*ALT* alanine aminotransferase, *AST* aspartate transaminase, *BUN* blood urea nitrogen, *HB* hemoglobin, *PLT* platelets, *PT* prothrombin time. A *P* < 0.05 = significant; *P* < 0.01 = highly significant

### Child–Pugh Scoring and Zinc Level Correlation

The results demonstrate a significant inverse correlation between serum zinc levels and the severity of liver disease, as assessed by the Child–Pugh classification. The Kruskal–Wallis H test revealed a statistically significant difference in mean serum zinc levels across the Child–Pugh scoring classes (*P* = 0.046). Mean serum zinc levels decreased progressively from 23.9 ± 26.2 µg/dL in class A to 22.8 ± 16.7 µg/dL in class B, and 7.9 ± 1.9 µg/dL in class C.

In terms of zinc status, the percentage of patients with clinical zinc deficiency (< 60 µg/dL) increased substantially with cirrhosis progression. Most patients in class A exhibited either normal zinc levels (41.67%) or marginal deficiency (41.67%), while clinical deficiency was less common (16.66%). In class B, the proportion of patients with clinical zinc deficiency (< 60 µg/dL) increased to 56.25%, while 41.25% had marginal deficiency, and only 2.5% maintained normal levels. By contrast, in class C, zinc deficiency was most pronounced, with 75% of patients exhibiting clinical deficiency, 25% showing marginal deficiency, and no patients maintaining normal zinc levels.

Spearman’s rank correlation analysis further confirmed a strong negative correlation between serum zinc levels and Child–Pugh class severity (*r* =  − 0.84, *P* < 0.001). These findings underscore the progressive depletion of zinc as liver disease worsens, particularly in advanced stages (class C), where zinc deficiency is ubiquitous. The observed trends highlight the clinical significance of serum zinc levels as a potential biomarker for liver disease severity and the need to monitor and address zinc status in patients with advanced liver disease (Table 4).

**Table 4:** Serum zinc levels across Child–Pugh scoring classes

Child–Pugh	Zinc category*	No. (%)^¥^	Mean serum zinc level (µg/dL)	Kruskal–Wallis (*P*-value)	Spearman’s *r* (*P*-value)
Class A	Normal Marginal deficiency Clinical deficiency	5 (41.67%) 5 (41.67%) 2 (16.66%)	23.9 ± 26.2	**0.046**	− 0.84 (< 0.001)
Class B	Normal Marginal deficiency Clinical deficiency	2 (2.5%) 33 (41.25%) 45 (56.25%)	22.8 ± 16.7		
Class C	Normal Marginal deficiency Clinical deficiency	0 (0.0%) 2 (25%) 6 (75%)	7.9 ± 1.9		

*The Japanese Society of Clinical Nutrition: deficiency (< 60 µg/dL), marginal deficiency (≥ 60 to < 80 µg/dL), and normal (≥ 80 µg/dL). ¥Percentages within groups

### Hepatic Encephalopathy Grades and Zinc Levels Correlation

The results demonstrate a significant inverse relationship between serum zinc levels and the severity of hepatic encephalopathy (HE), as assessed by the West Haven criteria. The Kruskal–Wallis H test revealed a statistically significant difference in mean serum zinc levels across HE grades (*p* = 0.024). Mean serum zinc levels declined progressively from 27.8 ± 26.5 µg/dL in Grade 1 to 17.0 ± 13.8 µg/dL in Grade 2, and 5.7 ± 0.2 µg/dL in Grade 3.

In terms of zinc status, the percentage of patients with clinical zinc deficiency (< 60 µg/dL) increased substantially with HE severity. In Grade 1, most patients (60.0%) exhibited clinical deficiency, while 30.8% had marginal deficiency and only 9.2% maintained normal zinc levels. In Grade 2, the prevalence of clinical deficiency rose to 50.0%, with 33.3% showing marginal deficiency and 16.7% retaining normal levels. By contrast, in Grade 3, most patients (80.0%) exhibited clinical deficiency, with only 20.0% showing marginal deficiency and no patients maintaining normal zinc levels.

Spearman’s rank correlation analysis confirmed a strong negative correlation between serum zinc levels, and HE grade (*r* =  − 0.78, *P* < 0.001). These findings highlight the progressive depletion of zinc with increasing HE severity, emphasizing the critical role of zinc deficiency in the pathophysiology of hepatic encephalopathy (Table 5).

**Table 5:** Serum zinc levels across hepatic encephalopathy grades (West Haven criteria)

HE grade (West Haven)	Zinc category*	No. (%)^¥^	Mean serum zinc level (µg/dL)	*Kruskal–Wallis* (*P*-value)	*Spearman’s r* (*P*-value*)*
Grade 1	Normal marginal deficiency Clinical deficiency	6 (9.2%) 20 (30.8%) 39 (600%)	27.8 ± 26.5	**0.024**	− 0.78 (< 0.001)
Grade 2	Normal marginal deficiency Clinical deficiency	5 (16.7%) 10 (33.3%) 15 (50%)	17.0 ± 13.8		
Grade 3	Normal marginal deficiency Clinical deficiency	0 (0.0%) 1 (20%) 4 (80%)	5.7 ± 0.2		

*The Japanese Society of Clinical Nutrition: deficiency (< 60 µg/dL), marginal deficiency (≥ 60 to < 80 µg/dL), and normal (≥ 80 µg/dL). ¥Percentages within groups

## Discussion

Zinc, the second most abundant intracellular trace element, is essential for many physiological functions. It acts as a cofactor for many enzymes and proteins involved in anti‑inflammatory, antioxidant, and apoptotic effects. It is also essential for cellular integrity and biological functions including the immune system, cognitive, reproductive, and cell growth and development. Zinc-binding proteins represent approximately 10% of human proteomes [28]. The liver plays an important role in zinc homeostasis; thus, zinc deficiency is often seen in individuals with chronic liver diseases. HE is one of the most common serious complications in decompensated liver cirrhosis/liver disease [29, 30].

In our study, the demographic homogeneity of the patient and control groups was demonstrated by the fact that there was no significant difference in the distribution of age or sex between the two groups. Variations in clinical outcomes can be linked to significant differences in clinical presentations. Complications of progressed liver dysfunction, including abdominal distension (72%), pedal edema (66%), and icterus (61%), were common in patients enrolled in the study. The systemic impact of decompensated cirrhosis is further highlighted by the predominance of additional neuropsychiatric symptoms, such as disorientation (47%) and asterixis (44%), which reflect early manifestations of hepatic encephalopathy. Furthermore, the high prevalence of hepatitis C (78%) and bilharzia (14%) as etiological factors underscores the regional uniqueness of cirrhosis in Egypt, necessitating targeted public health interventions.

These results are consistent with the known fact that decompensated cirrhosis causes severe systemic effects. It leads to portal hypertension, which disrupts blood flow and pressure. Inflammation and metabolic changes worsen the damage, often resulting in organ failure [31]. The clinical presentations reported in this study are consistent with prior findings about the most common symptoms of decompensated liver cirrhosis. Decompensated cirrhosis commonly presents with jaundice, ascites, leg edema, gastrointestinal bleeding, hepatic encephalopathy, and fatigue. These reflect severe liver dysfunction and multisystem involvement [32]. Neurological symptoms of chronic liver disease included hepatic encephalopathy, asterixis, seizures, movement disorders, cerebellar dysfunction, peripheral neuropathy, and spastic leg weakness, all caused by toxic buildup affecting the nervous system [33].

The etiological profile of cirrhosis in Egypt represents the unique health challenges in the region, shaped by the interplay between chronic viral infections, parasitic diseases, and lifestyle factors. Among the risk factors, the high prevalence of chronic hepatitis C virus infection is the major one; this has been one of the major public health issues resulting from past medical practices with unsatisfactory safety measures. Up to 13% of the population was infected with HCV, without or with cirrhosis or liver cancer [34]. In Egypt, the population prevalence of HBV was 1.4%, with an HBV-HCV co-infection rate of 0.06%. The nationwide vaccination program has decreased the prevalence of HBV infection considerably [35]. Egypt successfully implemented one of the world’s largest hepatitis C treatment campaigns, reducing new infections and cirrhosis cases by over half, but long-term infection burdens persist [36]. Schistosomiasis, a parasitic infection that is endemic in Egypt, causes liver fibrosis and cirrhosis, often aggravated by coexisting liver diseases [37]. Alcohol consumption, though lower than in many other parts of the world, still contributes to the overall burden of cirrhosis in Egypt [38]. Increasing obesity and diabetes rates have contributed to the increasing rates of metabolic dysfunction-associated fatty liver disease (MAFLD) [39]. Pesticide exposure and aflatoxins, especially in agricultural products, are significant risk factors for cirrhosis, with studies showing higher aflatoxin levels in patients with liver cancer [40]. New molecular research also uncovers genetic predispositions that influence cirrhosis development, paving the way for personalized treatments [41].

In this study, biochemical investigations showed significant derangement in liver, kidney, and hematological parameters among cirrhotic patients. High ALT, AST, total bilirubin, and direct bilirubin levels indicate hepatocellular damage and deranged bilirubin metabolism. Low albumin levels and prolonged prothrombin time (PT) indicate compromised synthetic function. Our study observed elevated BUN and creatinine levels in cirrhotic patients compared to controls, indicating potential renal dysfunction. Our findings also revealed significant reductions in hemoglobin and platelet counts in cirrhotic patients, while white blood cell counts showed no notable differences. Systemic fluid and electrolyte disturbances were represented in cirrhotic patients as indicated by reduced sodium levels, potassium, and calcium. Cirrhotic patients in our study displayed increased cholesterol, LDL, and HDL levels compared to controls, suggesting altered lipid metabolism. Zinc levels were significantly lower in the patient group (21.7 ± 24.3 µg/dL vs. 85.9 ± 32.6 µg/dL in controls, *P* < 0.0001), suggesting that zinc deficiency may be attributed to liver dysfunction as a complication of cirrhosis. However, our study did not find a significant difference in fasting blood glucose or HbA1c levels between patients and controls.

Our results align with previous studies that show significantly elevated aminotransferases (ALT and AST), hypoalbuminemia, and hyperbilirubinemia in cirrhotic patients. High levels of ALT and AST in cirrhotic patients reflect hepatocellular injury. Both these enzymes are released into the bloodstream after hepatocyte destruction. Although ALT is more specific for liver cells, AST may be elevated in many tissues; however, both are useful markers of liver inflammation or injury. In cirrhosis, due to chronic liver damage, there is a persistent elevation of ALT and AST, reflecting the severity of liver dysfunction [42]. Severe hypoalbuminemia and hyperbilirubinemia are indicative of liver dysfunction in cirrhotic patients. Hypoalbuminemia results from reduced hepatic synthesis of albumin, a crucial protein responsible for maintaining oncotic pressure and fluid balance. Hyperbilirubinemia is a result of impaired clearance of bilirubin by the liver, leading to the accumulation of bilirubin in the bloodstream. These abnormalities have become the hallmark of cirrhosis and a reflection of compromised liver function contributing to systemic complications such as edema, ascites, and jaundice [43, 44]. Several previously published studies have found altered kidney function markers in cirrhotic patients, with elevated BUN and creatinine levels often linked to hepatorenal syndrome (HRS) [45–47]. HRS is a severe complication of cirrhosis that arises from circulatory dysfunction because of the arterial vasodilation brought forth by portal hypertension and splanchnic vasodilation, leading to renal hypoperfusion along with systemic vasoconstriction. Imbalance in vascular tone results due to increased nitric oxide in the splanchnic circulation and due to the activation of the renin–angiotensin–aldosterone system, which notably contributed to acute kidney injury along with increased morbidity and mortality observed in cirrhotic patients [47].

Moreover, we observed disturbances in electrolytes and prolonged prothrombin time. The electrolyte disturbance and prolonged prothrombin time are common findings in cirrhotic patients, the result of impaired liver function. Such disturbances commonly include hyponatremia, hypokalemia, and hypocalcemia. Electrolyte imbalances among cirrhotic patients are commonly brought about by such factors as hepatorenal syndrome, ascites, and hypoalbuminemia. The liver, in this case, is unable to regulate fluid balance and produce necessary proteins, thus contributing to sodium and potassium imbalances. Moreover, the inability to synthesize proteins and clotting factors, along with gastrointestinal losses and diuretic therapy, further enhances such disturbances [48, 49]. Prolonged prothrombin time is due to a decline in the synthetic capability of the liver, relating to its reduced ability to synthesize clotting factors [50]. Our findings revealed significant reductions in hemoglobin and platelet counts in cirrhotic patients. Anemia and thrombocytopenia in cirrhosis occur due to multifactorial mechanisms. Anemia arises from gastrointestinal bleeding due to portal hypertension, nutritional deficiencies (iron, vitamin B12, folate), and hemolysis from hypersplenism. Thrombocytopenia develops from splenic sequestration secondary to splenomegaly, bone marrow suppression due to substances such as alcohol, and decreased thrombopoietin production secondary to the diseased liver. These abnormalities further increase the risk of bleeding and complicate the management of cirrhosis [51].

However, our study did not find a significant difference in fasting blood glucose or HbA1c levels between patients and controls. In cirrhotic patients, fasting blood glucose (FPG) and HbA1c levels often fail to reliably reflect true glycemic status due to the unique metabolic and hematologic changes associated with advanced liver disease. FPG may appear deceptively normal or only mildly elevated despite significant glucose intolerance, as cirrhosis impairs hepatic glucose metabolism and insulin clearance. Likewise, similarly low levels of HbA1c in cases of cirrhosis result from the short life of erythrocytes due to hypersplenism, hemolysis, or anemia. These factors all lead to low levels of HbA1c that inaccurately assess the patient’s long-term glycemic status [52]. The oral glucose tolerance test (OGTT) is the most reliable diagnostic tool for glucose intolerance or diabetes in cirrhosis, especially when FPG or HbA1c results are not conclusive in patients with disturbed metabolism [53]. The homeostasis model assessment of insulin resistance test (HOMA-IR) can be useful in research settings or as an adjunct for assessing insulin resistance but should be interpreted cautiously due to possible inaccuracies because of impaired insulin clearance in cirrhosis [54]. Moreover, postprandial glucose monitoring provides valuable insights into real-world glycemic fluctuation and is useful for maintaining glycemic control. Because cirrhotic patients frequently display aberrant glucose metabolism, including post-meal hyperglycemia, postprandial glucose monitoring is crucial. Compared to fasting measurements alone, this monitoring offers a more accurate evaluation of glucose control and identifies insulin resistance and beta-cell dysfunction in cirrhosis. It is recommended as a useful instrument for treating glucose imbalances in this group [55].

Our results showed, in conformity with several previous studies, significantly lower serum zinc levels among patients as compared to controls. Zinc deficiency is quite common in patients with cirrhosis of the liver, most studies showed significantly lower serum zinc in cirrhotic subjects than in healthy controls, and zinc deficiency increased with the severity of liver dysfunction [56–58]. Furthermore, reduced zinc levels have been implicated in the pathogenesis of hepatic encephalopathy, as zinc is a critical cofactor for enzymes involved in ammonia detoxification. Clinical research has also highlighted the potential therapeutic benefits of zinc supplementation in improving outcomes and mitigating complications associated with cirrhosis [59].

Observations show that many patients with liver cirrhosis are zinc deficient due to poor dietary intake, impaired absorption, and altered hepatic zinc metabolism caused by portal hypertension and gastrointestinal dysfunction in combination with increased urinary excretion later due to renal impairment [20]. Furthermore, the study highlights that reduced serum zinc levels correlate with the severity of cirrhosis. Zinc is involved in ammonia metabolism, as a cofactor for some of the urea cycle enzymes such as ornithine transcarbamylase and carbamoyl phosphate synthase. Therefore, zinc deficiency causes significant inefficient ammonia detoxification and leads to hyperammonemia, a common key feature in developing and worsening hepatic encephalopathy [60]. Zinc deficiency causes the condition to worsen in other ways. Zinc is required in antioxidant defense mechanisms, especially as a cofactor for enzymes like superoxide dismutase. Its deficiency increases oxidative stress, leading to greater hepatocyte injury and the release of pro-inflammatory cytokines, both of which aggravate liver tissue damage and propagate fibrosis by promoting the deposition of extracellular matrices [61]. Liver dysfunction due to cirrhosis impairs the body’s ability to utilize zinc effectively, which is critical in both the innate and adaptive immune systems and its deficiency impairs the body’s ability to mount an effective response to infections [62]. The clinical studies further support that zinc supplementation may improve clinical outcomes, particularly in managing complications like hepatic encephalopathy, and help mitigate some of the adverse effects seen in patients with chronic liver diseases [63].

In this study, serum zinc levels were significantly positively correlated with albumin levels (*r* = 0.843, *P* < 0.0001), emphasizing albumin’s role as the major transport protein for zinc in plasma. No correlations existed regarding zinc levels and other markers of liver function, including levels of bilirubin, creatinine, or platelet count. This suggests that hypoalbuminemia more closely relates to zinc deficiency than other evidence of liver dysfunction. This relationship also draws attention to zinc deficiency as a possible marker of nutritional and metabolic derangements in cirrhotic patients. No correlations existed between zinc levels and other clinical parameters of the study.

Our findings corroborate those of previous research findings which indicated that serum zinc level is positively correlated with serum albumin level [59, 60, 64, 65]. Such an association arises because, under physiological conditions, albumin is the major carrier protein for zinc during its transport through the bloodstream to various tissues and organs. When serum albumin concentrations are decreased, as they frequently are in liver disease, the binding and transport of zinc are likewise reduced, with a subsequent lowering of the serum concentration of this element [66, 67]. Furthermore, in a hypoalbuminemic state, the unbound or “free” zinc not bound to albumin becomes more labile and is excreted through the urine. This increased urinary zinc excretion exacerbates zinc depletion in those with low albumin levels [20]. All importantly, there is a clear association between zinc deficiency and acceleration in chronic kidney disease (CKD) progression. Low levels of serum zinc worsen kidney function by promoting oxidative stress, inflammation, and inappropriate ammonium metabolism [66]. Hypoalbuminemia enhances risks for renal injury. The findings imply that the amelioration of zinc deficiency, especially hypoalbuminemia, can delay the progression of both chronic liver and chronic kidney diseases. Thus, zinc supplementation could mitigate disease progression and improve clinical outcomes [63, 68].

The lack of significant correlations between zinc levels and renal parameters in cirrhosis patients in this study points out a peculiar feature of zinc deficiency in this condition. In contrast to other chronic diseases, where systemic inflammation or renal impairment often contributes to micronutrient disturbances, findings suggest that in cirrhosis, zinc deficiency is mainly driven by hepatic dysfunction, because the liver is responsible for its storage, metabolism, and synthesis of zinc-binding proteins like albumin [64]. This liver-specific etiology of zinc deficiency reinforces its potential role as a biomarker to reflect the degree of liver impairment and provide a non-invasive index of disease severity and therapeutic response. These findings emphasize a need for nutritional interventions, including zinc supplementation, targeting the cause of deficiency that is liver-related rather than systemic [69].

In this study, the mean serum zinc level decreased significantly with the increase in Child–Pugh classes, from 23.9 ± 26.2 µg/dL in class A to 7.9 ± 1.9 µg/dL in class C (*P* = 0.046). Indeed, zinc levels inversely correlate with the Child–Pugh score (*r* =  − 0.84, *P* < 0.001). The latter points toward the progressive depletion of zinc stores according to the progression of liver disease and puts the basis for including serum zinc levels as one of the non-invasive markers for staging the severity of cirrhosis. Our study also provided additional insight into the specific zinc deficiency thresholds across Child–Pugh classes, where it determined serum zinc levels as a specific cut-off as deficiency (< 60 µg/dL), marginal deficiency (≥ 60 to < 80 µg/dL), and normal (≥ 80 µg/dL), and stratified its distribution according to the Child–Pugh classes. Such stratification underscores progressive decline with advancing liver dysfunction and helps understand the relationship between deficiency of zinc and severity of cirrhosis. These thresholds provide an increased clinical value to the assessment of zinc levels, thus offering a clear framework for nutritional and metabolic impairment assessment in cirrhotic patients and appropriate management.

The Child–Pugh classification system has become one of the commonly used tools for stratification in distinct classes of severity within cirrhosis, serving well clinically in monitoring advancement and the prognosis of the disease course as patients progress to chronic liver failure. This classifies cirrhosis into three classes: well-compensated class A, partially compensated class B, and decompensated class C, where each provides a framework relevant to the distinct histological and morphological changes evident within the liver. This classification is important for clinical decision-making, the prognosis of disease course, and therapeutic intervention [70]. Previously published papers reported progressively lower zinc levels in advanced Child–Pugh classes [23, 58, 71, 72]. The serum zinc level decreases progressively in advanced classes of Child–Pugh, reflecting the worsening severity of liver dysfunction. This was supported by studies showing that zinc levels decreased significantly with an advance in Child–Pugh score, hence indicating a strong association of zinc deficiency with the advancement of liver disease. The lowest zinc levels are consistently reported in patients with decompensated cirrhosis, especially those classified under Child–Pugh class C. These findings indicate that zinc levels could be monitored as a marker of disease severity and support the hypothesis that hepatic dysfunction is the main cause of zinc deficiency in cirrhotic patients [23, 58].

In this study, serum zinc levels decreased progressively with worsening hepatic encephalopathy (HE) grades, from 27.8 ± 26.5 µg/dL in Grade 1 to 5.7 ± 0.2 µg/dL in Grade 3 (*P* = 0.024). The strong inverse correlation (*r* =  − 0.78, *p* < 0.001) highlights the potential role of zinc deficiency in HE pathogenesis, likely through impaired ammonia detoxification and neuronal dysfunction. Our study further clarified the relationship between zinc deficiency and the severity of HE by defining specific thresholds of zinc levels: deficiency (< 60 µg/dL), marginal deficiency (≥ 60 to < 80 µg/dL), and normal (≥ 80 µg/dL). In patients with Grade 3 HE, serum levels of zinc were almost totally depleted, thus indicating an important threshold below which, as suggested by the loss of metabolic functions, there may be irreversible brain injury. This finding underlines the importance of early administration of zinc to avoid the serious development of HE. Moreover, the absence of normal zinc levels in Grades 2 and 3 HE patients underlines that advanced cirrhosis may interfere with zinc metabolism and disturb liver and systemic functions. This points to the importance of targeted nutritional intervention for cirrhotic patients, especially those with deteriorating encephalopathy, to correct trace element imbalances.

The West Haven criteria represent a generally accepted system for the classification of the severity of HE in patients with liver cirrhosis, from Grade 1 (mild confusion, slight personality changes, and slight cognitive impairment), Grade 2 (Moderate confusion with disorientation to time and place), Grade 3 (severe confusion, marked disorientation, and significant cognitive dysfunction), to Grade 4 (coma, unresponsive). This grading allows the clinician to estimate the degree of cognitive and neurological dysfunction and provides a uniform means of diagnosing and monitoring the course of HE [73]. Recent studies have highlighted a significant association between serum zinc levels and the severity of hepatic encephalopathy (HE) in patients with liver cirrhosis, indicating that zinc deficiency is significantly associated with higher severity of cirrhosis and higher grades of HE [23, 74, 75]. These findings suggest that monitoring and addressing zinc deficiency could be crucial in managing HE in cirrhotic patients. Ammonia detoxification is greatly dependent on the presence of zinc, and its deficiency worsens HE due to impairment of the urea cycle. Zinc modulates gut microbiota, maintains intestinal barrier integrity, and diminishes the translocation of gut-derived toxins such as ammonia. Indeed, increased oxidative stress and exacerbated inflammation because of zinc deficiency contribute to worsening liver damage in cirrhosis. Supplementing zinc in cirrhotic patients with HE may improve cognitive functions, especially in covert HE, by restoring these highly critical biological processes [76, 77].

The strong correlation between serum zinc levels and the severity of liver cirrhosis and hepatic encephalopathy observed in this study reinforces the role of zinc as a critical trace element in the pathophysiology of these conditions. Lower zinc levels were significantly associated with worsening Child–Pugh classes and higher hepatic encephalopathy grades, highlighting zinc deficiency as both a marker of disease severity and a potential therapeutic target. Consistent with these findings, zinc deficiency has been linked to the progression of overt hepatic encephalopathy and increased mortality in patients with liver cirrhosis and minimal hepatic encephalopathy [78]. Moreover, the utility of simple biochemical screening models for detecting covert hepatic encephalopathy, as described in recent studies, offers a practical approach to improving early diagnosis and management [79]. Together, these findings underscore the importance of routine zinc monitoring, early intervention, and nutritional optimization as essential components of comprehensive care for cirrhotic patients to improve outcomes and prevent complications.

Unlike previous studies, this research uniquely evaluates serum zinc levels in an Egyptian cohort with cirrhosis primarily caused by hepatitis C and schistosomiasis. Our findings show significant correlations between serum zinc levels, Child–Pugh score, and hepatic encephalopathy (HE) grades, with progressively lower zinc levels observed across these clinical indicators. This study suggests that zinc deficiency serves as a marker for the worsening severity of cirrhosis and may offer a valuable clinical tool in guiding the management of liver disease. Additionally, we found a strong positive correlation between serum zinc and albumin levels, highlighting the potential role of hypoalbuminemia in exacerbating zinc deficiency in cirrhotic patients. These results provide new insights into nutritional and metabolic disturbances in cirrhosis and underline the importance of monitoring zinc levels in clinical practice.

Several limitations in this study need to be acknowledged. First, although the sample size was sufficient for statistical analysis, it may not allow firm conclusions to be made regarding wider populations with varied cirrhosis etiologies, especially those unrelated to hepatitis C or bilharzia. Second, serum zinc levels were measured as a single-point assessment and cannot reflect fluctuations influenced by dietary intake, medication use, or acute illness. Third, the ammonia level was not measured, which, if done, could give further direct clues on the role of zinc deficiency in the appearance of hepatic encephalopathy. Finally, this study did not evaluate the effects of zinc supplementation, which could be an area for future interventional research to assess its impact on hepatic encephalopathy severity and patient outcomes.

Future studies should be directed at longitudinal designs that assess the levels of zinc over time and their direct impact on clinical outcomes like survival, quality of life, and recurrence of encephalopathy. Interventional trials are needed to assess the efficacy of zinc supplementation in improving hepatic function, reducing ammonia levels, and alleviating the symptoms of encephalopathy. In addition, the interaction studies between zinc and other trace elements, such as copper or magnesium, may further demonstrate antagonistic or synergistic effects in the context of liver disease. The clinical significance of these results points out the importance of regular controls of zinc levels in patients with decompensated cirrhosis. Thereafter, establishing uniform guidelines for zinc supplementation in this population could significantly enhance treatment by addressing a potentially modifiable component of the disease process.

## Conclusion

Zinc deficiency has a strong correlation with the severity of liver cirrhosis and hepatic encephalopathy; serum zinc levels can act as a good biomarker for the assessment of disease severity. Regular evaluation of zinc levels in cirrhotic patients, especially those with hypoalbuminemia or advanced HE, is thus recommended. Supplementation of zinc deficiency and optimization of nutritional support may offer clinical benefits in improving patient outcomes.

## Data Availability

All relevant data are included in this published article.
